# Effects of Trimetazidine Pretreatment on Endothelial Dysfunction and Myocardial Injury in Unstable Angina Patients Undergoing Percutaneous Coronary Intervention

**DOI:** 10.1155/2019/4230948

**Published:** 2019-09-02

**Authors:** Shuai Shao, Zhaozhao Shi, Gary Tse, Xinghua Wang, Yanping Ni, Hongmei Liu, Tong Liu, Guangping Li

**Affiliations:** ^1^Tianjin Key Laboratory of Ionic-Molecular Function of Cardiovascular Disease, Tianjin Institute of Cardiology, Department of Cardiology, The Second Hospital of Tianjin Medical University, Tianjin 300211, China; ^2^Xi'An Number One Hospital, Xi'an 710002, China; ^3^Department of Medicine and Therapeutics, Chinese University of Hong Kong, Hong Kong SAR, China; ^4^Li Ka Shing Institute of Health Sciences, Faculty of Medicine, Chinese University of Hong Kong, Hong Kong SAR, China

## Abstract

**Objectives:**

Trimetazidine is an anti-ischemic medication licensed for the treatment of angina pectoris. However, the molecular mechanisms underlying its action remain incompletely elucidated. In this study, therefore, we examined the potential beneficial effects of trimetazidine on myocardial injury and endothelial dysfunction in patients with unstable angina in the perioperative period of percutaneous coronary intervention (PCI).

**Methods:**

A total of 97 patients with unstable angina were randomly divided into trimetazidine (*n* = 48) and control (*n* = 49) groups. All subjects received standard medical therapy. The trimetazidine group additionally received 20 mg trimetazidine three times daily 24 hours before and after PCI. Serum levels of creatine kinase-muscle/brain (CK-MB), cardiac troponin I (cTnI), heart-type fatty acid-binding protein (h-FABP), von Willebrand factor (vWF), and nitric oxide (NO) were measured before and the morning following PCI.

**Results:**

In the control group, levels of CK-MB, cTnI, and vWF were significantly elevated (*P* < 0.05) and NO level was decreased after PCI (*P* < 0.05). By contrast, no significant changes in the levels of these proteins were observed in the trimetazidine group after PCI (*P* > 0.05). Moreover, h-FABP levels were not significantly altered after PCI whether in the control or in the trimetazidine group (*P* > 0.05). Finally, a time-dependent increase in the levels of h-FABP from 0 to 6 hours after PCI, followed by a progressive decline, was observed (*P* < 0.05).

**Conclusions:**

PCI induces endothelial dysfunction and myocardial damage in patients with unstable angina. Trimetazidine therapy in the perioperative period can reduce this damage.

## 1. Introduction

Coronary artery disease (CAD) is a major cause of death in the developed world. Unstable angina belongs to the spectrum of presentations known collectively as acute coronary syndrome and is defined as myocardial ischemia without significant myocardial necrosis [1, 2]. Percutaneous coronary intervention (PCI) is an important management strategy for patients suffering from CAD [3]. However, complications such as postoperative myocardial injury and coronary in-stent restenosis remain unresolved problems, which negatively impact the prognosis [4]. Possible mechanisms of in-stent restenosis include physical damage to the endothelium, leading to the activation of proinflammatory pathways and thus neointimal hyperplasia, as well as chronic stent recoil [5].

Trimetazidine is a cytoprotective anti-ischemic agent that exerts its beneficial effects through several mechanisms that include inhibition of fatty acid oxidation, reduction in oxygen required for ATP production, and reduced intracellular accumulation of hydrogen ions, lactate, sodium ions, and calcium ions [6]. A previous systematic review and meta-analysis found that trimetazidine treatment was associated with reduced mean number of anginal attacks, fewer weekly nitroglycerin use, longer time for ST-segment depression to occur, higher total work, and capacity to undertake longer duration of exercise compared to other antianginal medications [7]. In a prospective, double-blinded study, trimetazidine was shown to have reduced endothelial dysfunction as reflected by improving both flow-mediated and nitroglycerin-induced dilatation of the brachial artery [8]. However, whether trimetazidine improves systemic markers of endothelial function remains unexplored. In this study, we examined the potential beneficial effects of trimetazidine on endothelial dysfunction and myocardial injury in patients with unstable angina in the perioperative period of PCI.

## 2. Materials and Methods

### 2.1. Study Details and Inclusion and Exclusion Criteria

The study was approved by the Second Hospital of Tianjin Medical University Ethics Committee. All subjects provided written, informed consent for enrolment into this study. A total of 97 patients with unstable angina presenting to our hospital from July to December 2017 were recruited. The inclusion criteria were as follows: (i) patients diagnosed with unstable angina pectoris according to established guidelines [9, 10], (ii) coronary angiography showing more than 75% stenosis in one or more coronary arteries, (iii) received PCI therapy, and (iv) provided informed consent. The exclusion criteria were subjects meeting any of the following criteria: (i) elevation of cTnI level >0.05 ng/L; (ii) intolerance to, or contraindicated for, trimetazidine, clopidogrel, or aspirin; those presenting with acute heart failure; and (iii) suffered from infections, liver and renal insufficiency, cancer, hemorrhagic disease, muscle injury, and underlying systemic diseases.

### 2.2. Treatment Groups

Patients were randomly divided into trimetazidine group (*n* = 48) and control group (*n* = 49) using the random seal envelope method. All subjects received standard medical therapy, which included aspirin, clopidogrel, or ticagrelor, low-molecular-weight heparin (LMWH), nitrates, rosuvastatin or atorvastatin, angiotensin-converting enzyme inhibitors (ACEI) or angiotensin receptor blocker (ARB), and/or *β*-receptor blocker. The trimetazidine group additionally received 20 mg trimetazidine three times daily at least 24 hours before PCI.

### 2.3. Specimen Collection and Detection

Peripheral venous blood (2 ml) was collected from all enrolled subjects immediately before PCI and at 6:00 AM the following morning after the procedure. The blood samples were subjected to centrifugation for 10 min at 2500 rpm/min. The sera supernatant separated was preserved at −80°C in a refrigerator. Detection of cTnI was performed using chemiluminescence immunoassay (Nanjing Jiancheng Biology Engineering Institute, Nanjing, China). CK-MB, h-FABP, and vWF were measured by ELISA (GBD, USA). NO was detected by the nitrate reductase method (Nanjing Jiancheng Biology Engineering Institute, Nanjing, China). All procedures were performed according to the protocols of the manufacturers.

### 2.4. Statistical Analysis

Statistical analysis was performed using SPSS software (version 19.0; SPSS Inc., Chicago, IL, USA). Descriptive statistics for continuous variables were expressed as mean ± standard deviation (*x̅* ± *s*), while the descriptive statistics for categorical variables were expressed as number (*n*) and percent (%). Analysis for normality of data (continuous variables) was performed using histogram of normal curve. Baseline differences between the two groups were analyzed using independent-sample *t*-test for continuous variables and chi-square test (*x*^2^) for categorical variables. Independent-sample *t*-test was used for comparisons of continuous variables between two study groups, whereas paired-sample *t*-test were used to compare before and after differences in means in group for continuous variables. One-way ANOVA was used for comparisons of time-dependent changes in the control group. A two-sided *P* < 0.05 was considered as statistically significant.

## 3. Results

### 3.1. Baseline Characteristics

In this study, a total of 97 patients (59 males and 38 females) with a mean age of 64.9 ± 10.8 years were included. Their baseline characteristics are shown in Table 1. No significant difference in age, gender, incidence in the comorbidities of hypertension, diabetes cerebral infarction or atrial fibrillation, smoking status, levels of total cholesterol, triglyceride, low-density lipoprotein cholesterol or high-density lipoprotein cholesterol, or left ventricular ejection fraction was observed between both groups (*P* > 0.05).

### 3.2. Markers of Myocardial and Endothelial Damage before and after PCI in the Control and Intervention Groups

**Figure 1:**
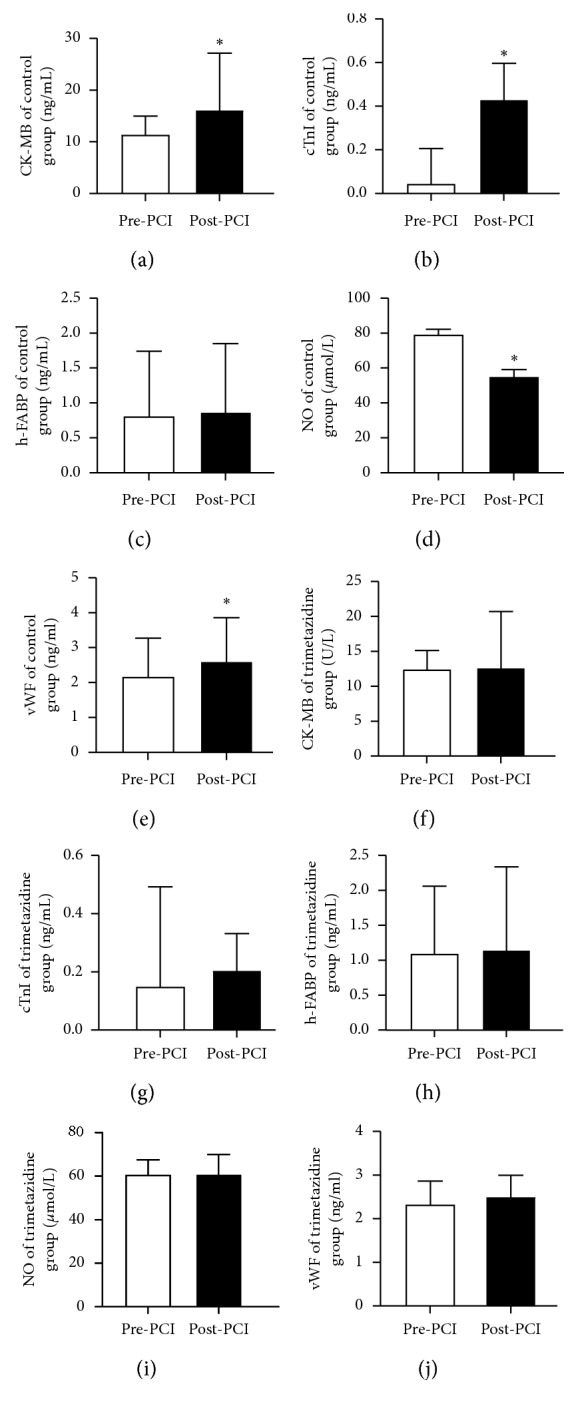
CK-MB, cTnI, h-FABP, NO, and vWF in the control and trimetazidine groups. ^*∗*^*P* < 0.05.

**Figure 2:**
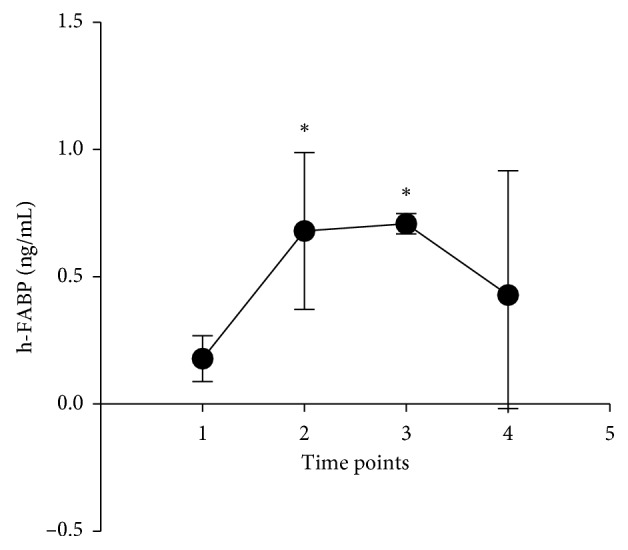
Levels of h-FABP at different time points for the control group. 1, pre-PCI right time; 2, 3 h post-PCI; 3, 6 h post-PCI; 4, >10 h post-PCI; ^*∗*^*P* < 0.05.

In the control group, the levels of myocardial injury, CK-MB (*P* < 0.05; Figure 1(a)) and cTnI (*P* < 0.05; Figure 1(b)) but not h-FABP (*P* > 0.05; Figure 1(c)), were significantly elevated after PCI (*P* < 0.05; values shown in Table 2). Moreover, for the endothelial markers, vWF was significantly increased (*P* < 0.05; Figure 1(d)) while NO level was significantly decreased (*P* < 0.05; Figure 1(e)) after PCI. By contrast, no significant changes in the levels of these proteins were observed in the trimetazidine group after PCI (*P* > 0.05; Figures 1(f)–1(j) and Table 3). The time-dependent changes in h-FABP were explored further. This revealed a progressive increase from 0 to 6 hours after PCI, followed by a decline thereafter compared to baseline in the control group (*P* < 0.05; Figure 2).

**Figure 3:**
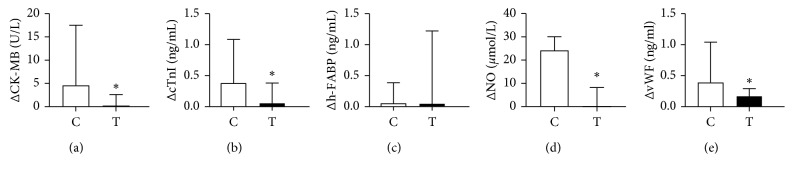
Difference in the levels of CK-MB, cTnI, h-FABP, NO, and vWF between pre- and post-PCI in the control and trimetazidine groups. C, control group; T, trimetazidine group; ΔCK-MB, the ascended levels of CK-MB; ΔcTnI, the ascended levels of cTnI; Δh-FABP, the ascended levels of h-FABP; ΔNO, the descended levels of NO; ΔvWF, the ascended levels of vWF; ^*∗*^*P* < 0.05.

Next, the changes in the levels of the above markers between the control and trimetazidine groups were compared (Table 4). For myocardial markers, the changes were significantly higher for CK-MB (*P* < 0.05; Figure 3(a)) and cTnI (*P* < 0.05; Figure 3(b)) but not for h-FABP (*P* < 0.05; Figure 3(c)). For endothelial markers, significantly greater changes in both NO (*P* < 0.05; Figure 3(d)) and vWF (*P* < 0.05; Figure 3(e)) were observed.

## 4. Discussion

The main findings of this clinical study are that in patients with unstable angina, trimetazidine treatment prevented the increase in CK-MB, cTnI, and vWF and the decrease in NO induced by PCI but did not significantly affect the levels of h-FABP.

ACS is a major cause of increased mortality in patients with CAD [11]. Its presentation ranges from unstable angina to non-ST elevation myocardial infarction (NSTEMI) and ST elevation myocardial infarction (STEMI). PCI is a common interventional approach for both alleviation of symptoms and reduction of coronary artery occlusion. However, the myocardial injuries that are induced by the procedure and the occurrence of in-stent restenosis are known complications of PCI [12]. PCI induces myocardial injury through the following mechanisms: (1) prolonged and repetition of balloon expansion as well as overextension; (2) occlusion of the coronary artery that occurs transiently during stent release [13]; and (3) ischemia-reperfusion injury that results from restoration of blood flow following periods of ischemia [14].

CK-MB and cTnI are established markers of myocardial injury, released by cardiomyocytes in response to damage. h-FABP is a low-molecular-weight protein (14.5 kDa) comprised of 132-amino acid residues found in cardiomyocytes [15]. Like CK-MB and cTnI, h-FABP is released rapidly into the bloodstream when these cells are damaged by acute ischemia [16, 17]. However, in our study, we found no significant changes in the levels of h-FABP, in contrast to increased CK-MB and cTnI after PCI. This prompted us to explore the time window over which h-FABP may change after PCI. Previous studies have shown that h-FABP was detected at high concentrations in blood samples within 1 hour of myocardial necrosis, and its concentration reached the maximum within 6–8 hours and generally decreased within 24–36 hours [18, 19]. In keeping with these findings, we found that h-FABP was significantly elevated within 3 hours, reaching peak levels at 6 hours, followed by a progressive decline after PCI. These in turn provided the explanation for the lack of changes detected 10 hours after PCI.

Moreover, vWF is stored in and secreted from the Weibel–Palade bodies (WPBs) of endothelial cells [20, 21]. It is an established cofactor in hemostasis, acting directly to recruit platelets to sites of vascular injury [20, 21]. It has thus been viewed as a biomarker of endothelial dysfunction, with high levels reflecting injured endothelium [22, 23]. Similarly, nitric oxide is the main endothelium-derived relaxing factor synthesized from L-arginine, oxygen, and NADPH by various nitric oxide synthase enzymes. Our results demonstrated higher vWF and lower NO levels after PCI in the control group, indicating the presence of endothelial injury caused by the procedure, which potentially underlies in-stent restenosis.

Previous studies showed that trimetazidine improves endothelial dysfunction and myocardial injury in patients with ischemic heart disease or chronic heart failure [8, 24, 25]. A meta-analysis including nine studies, with a total of 778 patients involved, reported that additional use of trimetazidine therapy was associated with reduction of serum of cTnI level [26]. Similarly, in our study, the increases in vWF, CK-MB, and cTnI and the decreases in NO were ameliorated by trimetazidine pretreatment before PCI. These findings suggest improvement in endothelial dysfunction and myocardial injury, possibly explained by the following actions. Firstly, trimetazidine is a selective inhibitor of mitochondrial long-chain 3-ketoacyl coenzyme A thiolase, reduces fatty acid *β*-oxidation, and increases the glucose oxidation within the myocardium, which results in a decrease in cellular acidosis, an increase in ATP production, and an improvement in cardiac efficiency, as well as an improvement in mitochondrial metabolism [27]. Secondly, trimetazidine optimizes cardiac metabolism, leading to an increased resistance to ischemic insults. Thirdly, trimetazidine reduces neutrophil accumulation and inhibits damage by free radicals [28, 29]. Finally, it alleviates intracellular calcium overload and vasospasm that occurs as a result of increases in free cytosolic calcium [25]. Together, these findings provide the mechanistic explanations for the protective effects of trimetazidine in both the endothelium and myocardium.

In this prospective, observational study, our results indicated the obvious advantages of additional use of trimetazidine before PCI, in line with data from a recently published study [30]. However, different from previous studies, this study aims at observing the protective effects of short-term preoperative preconditioning with trimetazidine on myocardial injury and endothelial function during the perioperative period of PCI. This discovery provides more new evidence for clinical treatment.

### 4.1. Limitations

Several limitations should be noted. Firstly, this study included a relatively small sample assessing changes in myocardial and endothelial injury markers over a short period of time. Further prospective studies are needed to explore the potential beneficial effects of trimetazidine over a longer timescale. Secondly, endothelial and myocardial damage was assessed using established biomarkers. Additional basic science studies are needed to better elucidate the underlying molecular mechanisms through which trimetazidine exerts its actions. Thirdly, given the benefit of statin pretreatment for patients with stable angina and acute coronary syndrome undergoing PCI [31], future studies can explore the potential synergistic effects of trimetazidine and statin pretreatment in this patient population. Fourthly, we did not conduct a subgroup analysis according to TIMI flow restoration and thrombus burden, which might also have effect on endothelial damage and myocardial injury. For the above limitations, more studies with large samples and rigorous design are needed in the future to provide more and better evidence for clinical treatment.

## 5. Conclusions

The endothelial and myocardial damage caused by PCI in patients with unstable angina can be prevented by perioperative use of trimetazidine.

## Figures and Tables

**Table 1:** Baseline characteristics of the control and trimetazidine groups.

Characteristics	Control group	Trimetazidine group	*P* values
Cases (*n*)	49	48	
Age (years)	64.22 ± 10.54	65.59 ± 12.07	0.399
Gender (male/female)	31/18	27/21	0.766
Hypertension (yes/no)	30/19	30/18	0.412
Diabetes (yes/no)	11/38	8/40	0.195
Cerebral infarction (yes/no)	4/45	8/40	0.240
Atrial fibrillation (yes/no)	2/47	1/47	0.845
Smoking (yes/no)	36/13	31/17	0.353
TC (mmol/L)	5.09 ± 1.08	4.86 ± 1.07	0.525
TG (mmol/L)	2.26 ± 1.92	1.78 ± 1.01	0.208
LDL-C (mmol/L)	2.94 ± 0.89	2.78 ± 0.64	0.627
HDL-C (mmol/L)	1.21 ± 0.38	1.12 ± 0.23	0.294
LVEF	0.56 ± 6.32	0.55 ± 7.83	0.822
Arterial stenosis (*n*)	2.26 ± 0.81	2.46 ± 0.73	0.389
Stent (*n*)	1.54 ± 0.74	1.68 ± 0.82	0.130
Blood collection time after PCI (*h*)	16.70 ± 2.46	16.68 ± 3.42	0.543

TC, total cholesterol; TG, triglyceride; LDL-C, low-density lipoprotein cholesterol; HDL-C, high-density lipoprotein cholesterol; LVEF, left ventricular ejection fraction.

**Table 2:** Serum levels of CK-MB, cTnI, h-FABP, NO, and vWF in the control groups, expressed as *x̅* ± *s*.

	CK-MB (U/L)	cTnI (ng/mL)	h-FABP (ng/mL)	NO (*μ*mol/L)	vWF (ng/ml)
Pre-PCI	11.25 ± 3.74	0.05 ± 0.18	0.84 ± 0.98	78.50 ± 3.75	2.18 ± 1.14
Post-PCI	16.02 ± 11.01	0.47 ± 0.19	0.90 ± 1.04	54.31 ± 4.97	2.62 ± 1.30
*P* values	0.043^*∗*^	0.000^*∗*^	0.333	0.000^*∗*^	0.005^*∗*^

PCI: percutaneous coronary intervention, CK-MB: creatine kinase-muscle/brain, cTnI: cardiac troponin I, h-FABP: heart-type fatty acid-binding protein, vWF: von Willebrand factor, and NO: nitric oxide.

**Table 3:** Serum levels of CK-MB, cTnI, h-FABP, NO, and vWF in the trimetazidine groups, expressed as *x̅* ± *s*.

	CK-MB (U/L)	cTnI (ng/mL)	h-FABP (ng/mL)	NO (*μ*mol/L)	vWF (ng/ml)
Pre-PCI	13.45 ± 3.06	0.16 ± 0.37	1.09 ± 0.97	60.53 ± 6.87	2.43 ± 0.58
Post-PCI	13.68 ± 8.87	0.22 ± 0.14	1.13 ± 1.21	60.46 ± 9.19	2.62 ± 0.54
*P* values	0.113	0.051	0.284	0.905	0.143

PCI: percutaneous coronary intervention, CK-MB: creatine kinase-muscle/brain, cTnI: cardiac troponin I, h-FABP: heart-type fatty acid-binding protein, vWF: von Willebrand factor, and NO: nitric oxide.

**Table 4:** Changes in CK-MB, cTnI, h-FABP, NO, and vWF levels before and after PCI in the control and trimetazidine groups, expressed as *x̅* ± *s*.

	ΔCK-MB (U/L)	ΔcTnI (ng/mL)	Δh-FABP (ng/mL)	ΔNO (*μ*mol/L)	ΔvWF (ng/ml)
Control group	4.77 ± 13.73	0.42 ± 0.78	0.06 ± 0.37	24.19 ± 5.85	0.43 ± 0.72
Trimetazidine group	0.23 ± 2.60	0.06 ± 0.37	0.04 ± 1.31	0.07 ± 7.90	0.19 ± 0.13
*P* value	0.016^*∗*^	0.035^*∗*^	0.640	0.010^*∗*^	0.002^*∗*^

ΔCK-MB: change in creatine kinase-muscle/brain, ΔcTnI: change in cardiac troponin I, Δh-FABP: change in heart-type fatty acid-binding protein, ΔNO: change in nitric oxide, and ΔvWF: change in von Willebrand factor. ^*∗*^*P* < 0.05.

## Data Availability

The data used to support the finding of this study are included within the tables of the article.
